# The Diaphragmatic Initiated Ventilatory Assist (DIVA) trial: study protocol for a randomized controlled trial comparing rates of extubation failure in extremely premature infants undergoing extubation to non-invasive neurally adjusted ventilatory assist versus non-synchronized nasal intermittent positive pressure ventilation

**DOI:** 10.1186/s13063-024-08038-4

**Published:** 2024-03-20

**Authors:** David N. Matlock, Sarah J. Ratcliffe, Sherry E. Courtney, Haresh Kirpalani, Kimberly Firestone, Howard Stein, Kevin Dysart, Karen Warren, Mitchell R. Goldstein, Kelli C. Lund, Aruna Natarajan, Ejigayehu Demissie, Elizabeth E. Foglia

**Affiliations:** 1 University of Arkansas for Medical Sciences, 4301 W. Markham St., Slot 512-5B, Little Rock, AR 72205 USA; 2 University of Arkansas for Medical Sciences, Little Rock, AR USA; 3 University of Virginia, Charlottesville, VA USA; 4 University of Pennsylvania Perelman School of Medicine, Philadelphia, PA USA; 5 McMaster University, Hamilton, ON Canada; 6 Akron Children’s Hospital, Akron, OH USA; 7 Ebeid Children’s Hospital, Toledo, OH USA; 8 Nemours Children’s Health Wilmington, Philadelphia, PA USA; 9 The Children’s Hospital of Philadelphia, Philadelphia, PA USA; 10 Loma Linda University School of Medicine, Loma Linda, CA USA; 11 University of Utah, Salt Lake City, UT USA; 12 National Heart, Lung and Blood Institute, National Institutes of Health, Bethesda, MD USA

**Keywords:** Respiratory distress syndrome of the neonate (RDS), Bronchopulmonary dysplasia (BPD), Non-invasive respiratory support, Patient-ventilator synchrony, Neurally adjusted ventilatory assist (NAVA), Nasal intermittent positive pressure ventilation (NIPPV)

## Abstract

**Background:**

Invasive mechanical ventilation contributes to bronchopulmonary dysplasia (BPD), the most common complication of prematurity and the leading respiratory cause of childhood morbidity. Non-invasive ventilation (NIV) may limit invasive ventilation exposure and can be either synchronized or non-synchronized (NS). Pooled data suggest synchronized forms may be superior. Non-invasive neurally adjusted ventilatory assist (NIV-NAVA) delivers NIV synchronized to the neural signal for breathing, which is detected with a specialized catheter. The DIVA (Diaphragmatic Initiated Ventilatory Assist) trial aims to determine in infants born 24^0/7^–27^6/7^ weeks’ gestation undergoing extubation whether NIV-NAVA compared to non-synchronized nasal intermittent positive pressure ventilation (NS-NIPPV) reduces the incidence of extubation failure within 5 days of extubation.

**Methods:**

This is a prospective, unblinded, pragmatic, multicenter phase III randomized clinical trial. Inclusion criteria are preterm infants 24–27^6/7^ weeks gestational age who were intubated within the first 7 days of life for at least 12 h and are undergoing extubation in the first 28 postnatal days. All sites will enter an initial run-in phase, where all infants are allocated to NIV-NAVA, and an independent technical committee assesses site performance. Subsequently, all enrolled infants are randomized to NIV-NAVA or NS-NIPPV at extubation. The primary outcome is extubation failure within 5 days of extubation, defined as any of the following: (1) rise in FiO_2_ at least 20% from pre-extubation for > 2 h, (2) pH ≤ 7.20 or pCO_2_ ≥ 70 mmHg; (3) > 1 apnea requiring positive pressure ventilation (PPV) or ≥ 6 apneas requiring stimulation within 6 h; (4) emergent intubation for cardiovascular instability or surgery. Our sample size of 478 provides 90% power to detect a 15% absolute reduction in the primary outcome. Enrolled infants will be followed for safety and secondary outcomes through 36 weeks’ postmenstrual age, discharge, death, or transfer.

**Discussion:**

The DIVA trial is the first large multicenter trial designed to assess the impact of NIV-NAVA on relevant clinical outcomes for preterm infants. The DIVA trial design incorporates input from clinical NAVA experts and includes innovative features, such as a run-in phase, to ensure consistent technical performance across sites.

**Trial registration:**

www.ClinicalTrials.gov, trial identifier NCT05446272, registered July 6, 2022.

## Administrative information

Note: the numbers in curly brackets as {} in this protocol refer to SPIRIT checklist item numbers. The order of the items has been modified to group similar items (see http://www.equator-network.org/reporting-guidelines/spirit-2013-statement-defining-standard-protocol-items-for-clinical-trials/)

**Table:** 

Title {1}	The Diaphragmatic Initiated Ventilatory Assist (DIVA) Trial: Study Protocol for a Randomized Controlled Trial
Trial registration {2a and 2b}.	www.clinicaltrials.gov, Trial identifier NCT05446272, Registered July 6, 2022.
Protocol version {3}	Protocol version 1.3, January 23, 2023
Funding {4}	This trial is funded by a grant from the National Heart, Lung, and Blood Institute (NHLBI). Grant number 1 UG3 HL152305-01A1 and U24 HL152304.
Author details {5a}	^2^University of Arkansas for Medical Sciences in Little Rock, AR, USA ^3^University of Virginia, Charlottesville, VA, USA ^4^University of Pennsylvania Perelman School of Medicine, Philadelphia, PA, USA ^5^McMaster University, Hamilton, ON, Canada ^6^Akron Children’s Hospital, Akron, OH, USA ^7^Ebeid Children’s Hospital, Toledo, OH, USA ^8^Nemours Children’s Health Wilmington, Philadelphia, PA, USA ^9^The Children’s Hospital of Philadelphia, Philadelphia, PA, USA ^10^Loma Linda University School of Medicine, Loma Linda, CA, USA ^11^University of Utah, Salt Lake City, UT, USA ^12^National Heart, Lung and Blood Institute, National Institutes of Health, Bethesda, MD, USA
Name and contact information for the trial sponsor {5b}	The lead clinical site (sponsor) is the University of Pennsylvania. The trial is funded by the NHLBI.
Role of sponsor {5c}	The lead clinical site (sponsor) is the University of Pennsylvania. The trial investigators have oversight of all aspects of trial design and execution.

## Introduction

### Background and rationale {6a}

Bronchopulmonary dysplasia (BPD) is the most common complication of prematurity and remains the leading respiratory cause of childhood morbidity [1–11]. BPD is an important determinant of the long-term neurodevelopmental outcome of infants and has societal impacts, including a cost of over $2.4 billion per annum in the United States [10–13]. Advances in neonatal care have led to increased survival among extremely premature infants (born less than 28 weeks’ gestation or 1000 g). Despite improved survival, BPD rates remain high [2].

Animal and clinical studies demonstrate that ventilator-induced lung injury (VILI) is a major factor in the development of BPD [14]. Both oxygen and positive pressure ventilation injure the immature preterm lung and cause VILI [15, 16]. Because even brief exposure to intubated positive pressure ventilation is injurious, avoiding invasive mechanical ventilation is the most widely acknowledged strategy to prevent VILI and BPD. Therefore, time on ventilators and rates of successful extubation are important endpoints of therapy.

Currently, no consensus evidence-based approaches exist to identify infants who can be successfully extubated. Therefore, extubation failure occurs in as many as 50% of infants [17, 18]. Exposure to multiple courses of mechanical ventilation is common and associated with increased risk for BPD [19, 20]. Intubation is a high-risk procedure, with adverse events occurring in 19% and severe events in 4% [21]. Recurrent extubation/reintubation risks airway damage and is associated with subglottic stenosis [22]. Therefore, preventing extubation failure requiring reintubation is a clinically relevant target to prevent acute and chronic airway and lung injury in extremely preterm infants [23–25].

The Diaphragmatic Initiated Ventilatory Assist (DIVA) trial is an unblinded, pragmatic, multicenter phase III randomized clinical trial in extremely preterm infants 24^0/7^–27^6/7^ weeks gestational age to determine if non-invasive neurally adjusted ventilatory assist (NIV-NAVA), compared with non-synchronized nasal intermittent positive pressure ventilation (NS-NIPPV), prevents extubation failure within 5 days (120 h) of extubation from mechanical ventilation.

### Objectives {7}

The primary objective is to compare rates of extubation failure within 5 days of extubation between extremely preterm infants treated with NIV-NAVA to those treated with NS-NIPPV.

The secondary objective is to compare respiratory and safety outcomes through 36 weeks postmenstrual age (PMA) among infants treated with NIV-NAVA vs. NS-NIPPV.

### Trial design {8}

The DIVA trial is a prospective, pragmatic, multi-site, phase III randomized trial to investigate the effectiveness of NIV-NAVA vs. NS-NIPPV in preventing extubation failure within 5 days of extubation. After sites demonstrate proficiency managing NIV-NAVA during a run-in phase of 3–10 infants per site, infants will be randomized to receive either NIV-NAVA or NS-NIPPV following extubation, with a primary outcome of extubation failure within 5 days of extubation. The randomization phase uses a two-arm group sequential design (GSD) with two interim looks. Treatment allocation is blinded, though implementation of intervention is not blinded. Infants enrolled during the run-in phase will not be included in the primary or secondary outcomes analysis.

## Methods: participants, interventions, and outcomes

### Study setting {9}

The study will occur in neonatal intensive care units in the United States and Canada. A list of participating sites is available at https://clinicaltrials.gov/.

### Eligibility criteria {10}

Inclusion criteria:

- Gestational age of 24–27^6/7^ weeks at birth
- Intubated in the first 7 days of life
- Undergoing extubation following at least 12 h of invasive mechanical ventilation
- Postnatal age < 28 days at the time of extubation

Exclusion criteria:

- Major congenital anomalies, including pulmonary hypoplasia
- Neurologic disorders affecting respiratory drive (other than apnea of prematurity)
- Esophageal bleeding or other contraindications to naso/orogastric catheter placement
- Current weight < 500 g (based on Edi catheter approval)
- Study ventilator not available at time eligibility criteria are met
- Planned surgery or invasive procedure within 5 days of extubation
- Informed consent not provided

### Who will take informed consent? {26a}

A member of the study team will approach parents to offer study participation and obtain informed consent. A DIVA trial educational video was developed to augment this discussion and allow families to review key information about the trial. https://youtu.be/zqzZYImXaHU

### Additional consent provisions for collection and use of participant data and biological specimens {26b}

The informed consent form states that participant data may be used to support secondary research analyses. A separate informed consent will be obtained for any ancillary studies that require additional research procedures. No biological specimens are collected in the trial.

## Interventions

### The explanation for the choice of comparators {6b}

Meta-analysis favors NIPPV over continuous positive airway pressure (CPAP) to prevent extubation failure in preterm infants. Data from clinical trials suggest that synchronized support is beneficial, but effective methods of achieving this synchrony have been limited until recently. Preliminary data of extubation practice at the original DIVA trial study sites showed an equal use of NIV-NAVA (35%) and NS-NIPPV (38%) for preterm infants (remainder CPAP or high-flow nasal cannula) (Table 1). These data confirm clinical and ethical equipoise for the control intervention at participating sites.

**Table 1:** Extubation failure rates within 5 days for infants 24–27^6/7^ weeks gestational age at DIVA trial sites

Mode	Extubation failure		
	Yes	No	Total
NIV-NAVA	7 (18.9%)	30 (81.1%)	37
NIPPV	17 (40.5%)	25 (59.5%)	42

### Intervention description {11a}

**Fig. 1:**
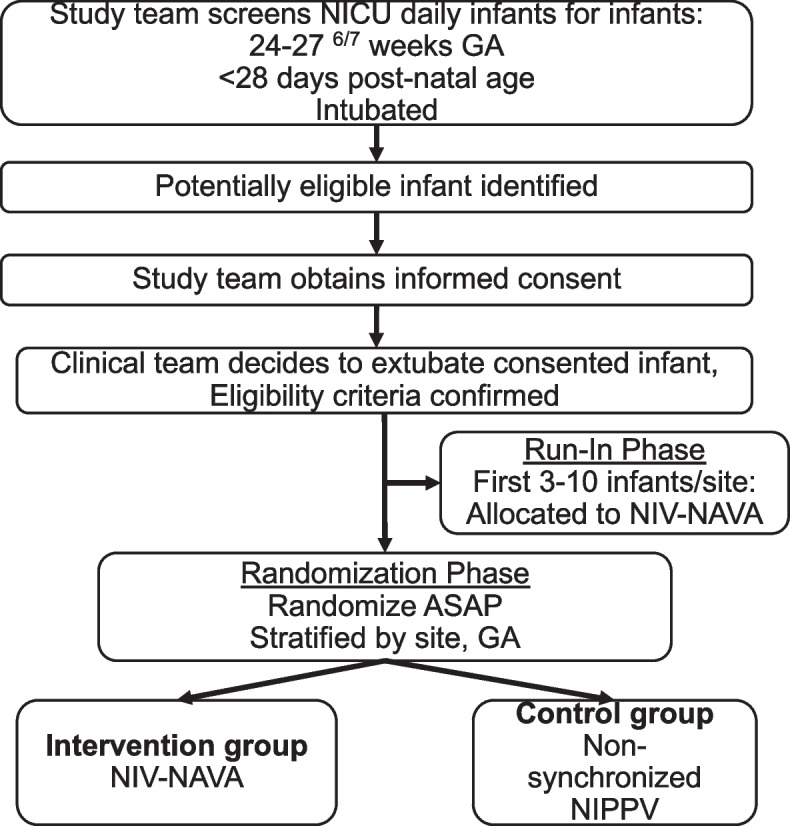
Flow diagram for screening, consent, and treatment allocation for DIVA trial. NICU, neonatal intensive care unit; GA, gestational age; NIV-NAVA, non-invasive neurally adjusted ventilatory assist; ASAP, as soon as possible; NIPPV, nasal intermittent positive pressure ventilation

There are 2 phases in the trial. First is an initial run-in phase, in which all enrolled (consented) and eligible infants at each site are allocated to NIV-NAVA to ensure site-level baseline NIV-NAVA technical proficiency and protocol adherence. Following this phase, sites progress to the randomization phase, where all enrolled infants are randomly allocated in a 1:1 fashion to either NIV-NAVA or NS-NIPPV (Fig. 1).

### Respiratory management

Protocol-mandated aspects of respiratory management during the intervention period include the following:

- Infants will be managed according to their allocated support mode for 5 days (120 h) after extubation, with no cross-over between groups
- NIV-NAVA will be delivered using the Servo-I, Servo-N, or Servo-U with the associated Edi catheter. All of these have FDA clearance for neonates weighing > 500 g
- Infants in the control arm will be managed using an FDA-approved respiratory device, per the study unit’s standard of care
- For both treatment groups, respiratory support will be provided using an interface with FDA approval or clearance to provide continuous positive pressure for preterm infants. RAM cannula and other non-approved interfaces, therefore, will not be used
- All infants must be treated with systemic (enteral or parenteral) caffeine throughout the study intervention period
- Ventilator settings will be titrated according to local unit norms within the allowable DIVA protocol settings (Table 2). These reflect variations in site practice across all DIVA trial sites. Sites do not need to escalate parameters to the maximal values provided. Guidelines for escalating and weaning ventilator support in both arms are provided in the manual of operations

**Table 2:** Range of allowable NIV-NAVA and NS-NIPPV settings

	**Intervention arm: NIV-NAVA**	**Control arm: NS-NIPPV**
PEEP	5–10 cm H_2_O	5–10 cm H_2_O
PIP	N/A, backup PIP 15–30 cm H_2_O	15–30 cm H_2_O
Rate	N/A, backup rate 10–60	10–60

### Criteria for discontinuing or modifying allocated interventions {11b}

Study infants should remain on the allocated mode of support throughout the entire 5-day intervention period. However, if an infant has been weaned to minimal settings for the allocated mode of support with FiO_2_ 21% for at least 12 h, they may be weaned to CPAP per clinical judgement. Weaning an infant to CPAP before meeting these criteria for at least 12 h is a protocol violation.

### Strategies to improve adherence to interventions {11c}

Every effort will be made to allocate and initiate study treatment for enrolled (consented) and eligible infants as close to the time of extubation as possible, with no more than 2 h between extubation and initiation of allocated respiratory therapy. In the case of an unplanned extubation for an infant with informed consent, if the clinical team determines to provide a trial of non-invasive support, the infant will be allocated to study treatment as soon as possible, no later than 2 h after extubation.

### Internal monitoring

The site principal investigator (PI) will be responsible for internally monitoring each infant meeting the extubation failure criteria. The site PI will review the ventilator management for that infant and confirm whether ventilator settings were adjusted appropriately per unit norms and within the allowable DIVA trial parameters.

### External monitoring

The DIVA technical committee will assess all sites’ technical performance during the trial for infants on both NIV-NAVA and NS-NIPPV. During the run-in phase, each site’s performance with NAVA will be assessed using ventilator screenshots respiratory settings data (Table 3). Sites will not be able to randomize infants until NAVA proficiency is demonstrated. In the randomization phase, sites will continue to monitor and evaluate their performance in both arms of the study. The technical committee will monitor the first six randomized infants, and a random 10% will be monitored thereafter. The technical committee will use the run-in phase approach to monitor infants treated with NIV-NAVA in the randomization phase (Table 3). In addition, NIPPV settings and clinical data will also be evaluated systematically for infants randomized to NS-NIPPV.

**Table 3:** NIV-NAVA ventilator screen shots assessed by technical committee

Parameter assessed	Interval
Edi catheter screen	Once
Alarm limit screen	Once
Time in back up and FiO_2_	3-h trend
Time in back up and FiO_2_	24-h trend
Edi peak, PIP, respiratory rate	3-h trend
Edi peak, PIP, respiratory rate	24-h trend
Ventilator setting/parameter screen	Once

### Relevant concomitant care permitted or prohibited during the trial {11d}

Beyond specified protocol-mandated aspects of care, infants will be managed according to local clinical protocols for respiratory medications and titrating supplemental FiO_2_ to maintain local SpO_2_ targets. The executive committee will determine whether concurrent enrollment in other site studies is acceptable.

### Provisions for post-trial care {30}

Enrolled infants will be managed as directed by the local clinicians following the 5-day intervention period. Secondary and safety outcomes will be ascertained until 36 weeks’ PMA, death, or discharge (whichever comes first).

## Outcomes {12}

### Primary study endpoint

The primary study endpoint is extubation failure. Extubation failure is defined when an infant is on the allocated mode of respiratory support and meets any of the following four criteria: (1) rise in FiO_2_ at least 20% from pre-extubation value for > 2 h to maintain local SpO_2_ targets, (2) pH ≤ 7.20 or pCO_2_ ≥ 70 mmHg; (3) > 1 apneic event requiring positive pressure ventilation (PPV) within 6 h or ≥ 6 apneic events requiring stimulation within 6 h; (4) emergent intubation by the clinical team for cardiovascular instability or surgery; (5) any other intubation.

### Rationale for primary endpoint

Extubation failure is a clinically relevant short-term outcome consistent with VILI’s etiological role in BPD. However, there are no universally accepted criteria for intubation in this population. We therefore define the primary outcome as extubation failure using objective clinical physiological criteria rather than a practice-based outcome such as re-intubation, which could be susceptible to performance bias in this unblinded trial. This is consistent with other high-quality trials of respiratory management in the neonatal literature [26–28].

### Secondary study endpoints

The DIVA trial is not powered to identify the impact of NIV-NAVA on all later respiratory outcomes. However, exploratory analyses of the impact of NIV-NAVA on respiratory outcomes and the association between timing and occurrence of extubation failure on longer-term respiratory morbidity among extremely preterm infants will be conducted. Enrolled infants will be followed until 36 weeks PMA for the following secondary outcomes:

- Bronchopulmonary dysplasia at 36 weeks PMA, as defined by Jensen et al. [29]
- Composite of death/BPD
- Endotracheal intubation, including time and reason for intubation
- PMA at last mechanical ventilation, last positive pressure, and last supplemental oxygen
- Post-randomization postnatal steroids
- Brain injury (intraventricular hemorrhage and periventricular leukomalacia)
- Patent ductus arteriosus requiring therapy
- Pulmonary hemorrhage
- Culture proven sepsis
- Necrotizing enterocolitis
- Retinopathy of prematurity
- Time to death
- Air leaks
- Gastrointestinal perforation or bleeding

## Participant timeline {13}

### Duration of study participation

**Fig. 2:**
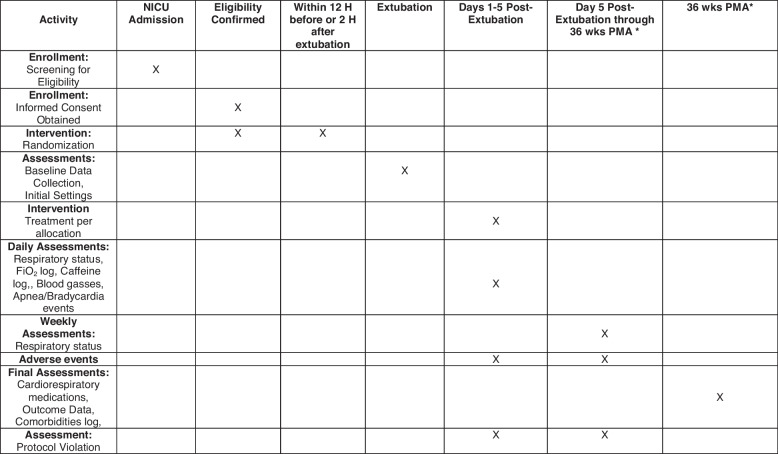
Schedule of enrollment, interventions, and assessments (SPIRIT figure). Abbreviations: FiO_2_, fraction of inspired oxygen; H, hours; PMA, post-menstrual age; wks, weeks. Asterisk symbol (*) indicates the following: for run-in phase, all enrolled infants are allocated to NIV-NAVA at randomization and data collection ends after 5 days of extubation

Figures 1 and 2 outline the timing of enrollment, trial interventions, and data collection. Subject participation will commence upon enrollment following informed consent in the trial, as early as 24 weeks PMA, and will continue until all secondary outcomes are ascertained by 36^6/7^ weeks PMA. Thus, the total duration of study participation will be up to 13 weeks for each study subject.

### Sample size {14}

The calculated study sample size accounts for 478 infants in the randomization phase. Very little published data exists for reintubation rates using NIV-NAVA or NS-NIPPV in our study population. In a recent pilot RCT of more mature preterm infants (median 27 weeks’ gestation), 9% of infants managed with NIV-NAVA were reintubated within 5 days [30]. Our preliminary data indicates a higher failure rate in our more immature study population (Table 1). Sample size estimates were based on having 90% power to detect a 15% absolute risk reduction in extubation failure among infants treated with NIV-NAVA, compared with non-synchronized NIPPV, assuming a two-sided test, family-wise error rate of 0.05 and a group sequential design with 2 interim analyses. The interim analyses were set after 50% and 75% of the infants have completed the primary outcome and used Len-DeMets spending functions of the O’Brien-Fleming type. Simulation studies were used to determine that interim analyses prior to 50% completion of the primary outcome were highly unlikely to result in any clinically meaningful differences in the primary efficacy and safety outcomes while reducing power for the final analysis.

Preliminary data from the DIVA trial sites informed sample size calculations: following extubation, 40% of infants treated with NS-NIPPV were reintubated within 5 days, and 19% of infants treated with NIV-NAVA were reintubated within 5 days (Table 1). This reintubation rate for NS-NIPPV is consistent with the reintubation rate of 37% among infants treated with NIPPV in the largest trial of NIPPV vs. CPAP (*n* = 1009) [19].

Thus, we conservatively anticipate a 5-day extubation failure rate of 35% in the NS-NIPPV group. We assume *90% power* and a two-sided proportions test. To detect a 15% absolute reduction in the rate of extubation failure rate in the NIV-NAVA group would require 406 infants. We expect 25% of enrolled infants to be from a multiple birth (based on the PIs’ previous trials and published papers). The multiple birth design effect is 1.15, assuming an intraclass correlation coefficient of 0.6. Inflating the required sample size for the design effect and conservatively allowing for 2% drop-out prior to the primary outcome results in 478 randomized infants (Table 4).

**Table 4:** Sample size requirements for the primary outcome

Power	Failure rate in non-synchronized NIPPV arm	Failure rate in NIV-NAVA arm	GSD sample size	ICC	Design effect	Sample size (total infants randomized)
90%	30%	15%	358	0.4	1.10	402
				0.6	1.15	422
		20%	844	0.4	1.10	948
				0.6	1.15	992
	35%	15%	222	0.4	1.10	250
				0.6	1.15	262
		20%	406	0.4	1.10	456
				0.6	1.15	478
	40%	15%	154	0.4	1.10	174
				0.6	1.15	182
		20%	246	0.4	1.10	278
				0.6	1.15	290

Only infants in the randomization phase will be included in the analysis of NIV-NAVA vs. NS-NIPPV for specified outcomes; infants from the run-in phase do not contribute to the analysis. We anticipate enrolling (consenting) up to two infants for every infant who meets all eligibility criteria and is allocated to treatment, as not all consented subjects will be eligible for the trial at the time of extubation. Thus, we expect to enroll (consent) up to 1080 infants to obtain 540 eligible subjects allocated to study treatment across both phases.

### Recruitment {15}

All study participants will be inpatients in the neonatal intensive care unit (NICU) at sites experienced in clinical trials. These sites have existing screening and consent procedures for neonatal trials, which include daily queries of new NICU admissions to identify and follow potentially eligible subjects. Parents of potentially eligible infants will be approached by a member of the clinical study team as soon as possible after birth to offer study participation, allowing for treatment allocation at the time of later clinical extubation if within the first 28 days of life.

## Assignment of interventions: allocation

### Sequence generation {16a}

An online, electronic data management system (DMS) randomization module, including a randomization form, will generate the sequence for randomization, maintaining a balance between treatment arms using randomly permutated blocks. Randomization is stratified by site, gestational age group (24–25^6/7^, 26–27^6/7^ weeks), and time on mechanical ventilation prior to extubation (≤ 7 days, > 7 days), in a 1:1 allocation. Twins and multiples will be randomized individually. Each participant will only be randomized once.

### Concealment mechanism {16b}

In order to protect allocation concealment, randomization occurs as close as possible to the clinical decision to extubate an eligible infant (or to provide a trial of non-invasive support after unplanned extubation). Allocation is blinded prior to randomization.

### Implementation {16c}

The DMS randomization module requires site staff to enter eligibility confirmation data to verify the appropriateness of randomization. In particular, the availability of a ventilator capable of providing either support mode will be confirmed before randomization occurs. Once infant eligibility and equipment availability are confirmed, the module will perform the randomization, record the assignment to the subject, and display the treatment arm on the screen for the site staff.

## Assignment of interventions: blinding

### Who will be blinded {17a}

The study will be blinded in treatment allocation; patients, parents, and clinicians will be unblinded once treatment is assigned, and then, they are blinded to the analyses. Since this technology is visible, this trial is unblinded to the infant’s clinical team.

### Procedure for unblinding if needed {17b}

This trial is unblinded. Analyses will be unblinded once all outcome comparisons are completed.

## Data collection and management

### Plans for assessment and collection of outcomes {18a}

#### Study measures

Study measures will be ascertained from the maternal and infant medical records or collected from ventilator logs. Key data to be collected for all subjects allocated to treatment are listed in Table 5.

**Table 5:** Key data to be collected for enrolled subjects

Topic and timing	Specific data elements
Pre-randomization characteristics	*Demographics*: birth weight, gestational age at birth, sex, race, ethnicity, multiple gestations, small for gestational age at birth, postnatal age at randomization *Maternal characteristics*: antenatal steroids, clinical diagnosis of chorioamnionitis, diagnosis of PPROM, mode of delivery *Postnatal interventions*: surfactant (number, timing, and method of administration), postnatal steroids *Respiratory interventions*: duration of mechanical ventilation, mode of ventilation and highest settings, ventilator settings at the time of extubation, postnatal age (days) at extubation, interval (hours) between extubation and initiation of allocated ventilator mode
Daily respiratory status, obtained daily for the first 5 days after treatment allocation	*Extubation failure criteria*: blood gas values, highest FiO_2_, number of apnea/bradycardia events over the previous 24 h Intubation, including timing and indication All respiratory settings and FiO_2_ values
Respiratory Status obtained weekly from treatment allocation until 36 weeks PMA	Timing and indication for all intubations Timing of extubation Start/stop dates of modes of support (i.e., invasive ventilation, non-invasive ventilation, CPAP, cannula, room air) Respiratory medications, including diuretics and postnatal steroids PMA at last invasive ventilation, positive pressure, and supplemental oxygen
Respiratory status at 36 weeks PMA	Respiratory support mode and settings at 36 weeks PMA Supplemental oxygen at 36 weeks PMA
Safety outcomes by 36 weeks PMA	Air leaks, GI perforation and bleeding, and death (date and primary cause of death; narrative autopsy results if performed)
Secondary clinical outcomes by 36 weeks PMA	Brain injury, retinopathy of prematurity, sepsis, patent ductus arteriosus, pulmonary hemorrhage, necrotizing enterocolitis Any surgical procedures

### Plans to promote participant retention and complete follow-up {18b}

The study follow-up phase will commence after primary outcome ascertainment and continue until 36 weeks PMA, death, or hospital discharge/transfer (whichever is first). Only randomized patients will be followed (there will be no follow-up for patients in the run-in phase).

For subjects whose parents withdraw consent to participate in the study, the study team will request permission for ongoing collection and use of study-related measures and endpoints from the medical record*.*

### Data management {19}

Project and data management support will be provided by the research team at the University of Pennsylvania Clinical Research Computing Unit and biostatisticians at the University of Virginia (UVA). Initial stages of data management will be done by experienced clinical data managers and database developers, supported by computer system analysts, programmers, and information technology specialists. These personnel will be responsible for data quality and timeliness, documentation of processes and procedures, and training of data management staff.

### Data security

Each site will maintain its screening logs with identifiable information. Only dates (from protected health information) will be shared with the data coordinating center (DCC). Each site will directly enter all data into the securely maintained DCC REDCap DMS.

### Confidentiality {27}

The DIVA study confidentiality procedures are designed to protect potential participants and ensure the confidentiality, security, and integrity of all data collected for this trial.

### Plans for collection, laboratory evaluation, and storage of biological specimens for genetic or molecular analysis in this trial/future use {33}

The trial involves no plans for collection, laboratory evaluation, and storage of biological specimens for genetic or molecular analysis.

## Statistical methods

### Statistical methods for primary and secondary outcomes {20a}

The proposed study is a two-arm parallel design of two alternative courses of treatment. A modified “as-randomized” analysis (or “intention-to-treat” analysis) (mITT) will estimate whether NIV-NAVA is superior to NS-NIPPV in avoiding treatment failure and is at least as good as NS-NIPPV in avoiding adverse events.

Among infants randomized before extubation, there is a risk that infants will become ineligible for extubation (and from the trial) after randomization due to changes in clinical status. Therefore, a mITT analysis has been planned. Only infants whose eligibility changes due to new/emergent conditions not known at the time of randomization will be excluded from the primary outcome analysis set in the mITT analysis. This number is expected to be minimal and will be closely monitored. A sensitivity analysis using a conventional ITT analysis will also be conducted.

The primary outcome will compare rates of extubation failure between the treatments using logistic regression. The regression model will be fitted using robust clustered standard errors under the generalized estimating equation (GEE) framework, with an exchangeable correlation structure for clusters based on infant mother, i.e., adjusting for the inherent correlation between infants that are multiples. The primary analysis will include clinical site, gestational age, biological sex, and days of invasive ventilation prior to extubation as fixed effects, to control for any residual confounding and site-level treatment imbalance from the stratification factors. Standard regression diagnostics will be used to assess model adequacy and to examine for potential outlying or influential data points.

Secondary efficacy and safety outcomes will be analyzed using similar procedures to the primary outcome. Comparisons between treatment arms will use GEE based logistic regression (dichotomous outcomes), linear regression (continuous outcomes), Poisson regression (count outcomes), or survival analysis (time based outcomes), as appropriate.

### Interim analyses {21b}

Two interim analyses are planned for the primary outcome of extubation failure: (1) after 50% and (2) 75% of the subjects have completed the primary outcome.

### Methods for additional analyses (e.g., subgroup analyses) {20b}

Prior studies offer no basis for assuming a priori interactions between treatment arms and subgroups defined by sex, race/ethnicity, gestational age, site or a combination of these groups, beyond that already controlled for in the randomization. For that reason, preplanned tests for interactions with treatment assignment are not warranted, and not powered for. We will, however, table all results by subgroups for descriptive purposes and to explore in secondary analyses possible subgroup differences by treatment group, solely for purposes of generating hypotheses for future studies. The subgroups will include sex, race/ethnicity, gestational age, site, maternal corticosteroid use, reason for initial intubation, and days intubated prior to extubation attempt. Efficacy in NAVA naïve versus non-naïve sites will be compared. Subgroup analyses will use either permutation tests or regression models (GEEs) with a subgroup-arm interaction term based on subgroup sample size and distributional properties of the randomization scheme in the subgroup.

For the 36 weeks’ PMA outcomes, evaluating the interaction between the primary outcome, treatment arm, and reintubation rates will be important for future clinical trial design. Subgroup analyses exploring these interactions will be performed for all outcomes evaluated after the primary outcome. For non-mortal 36 weeks’ PMA/discharge secondary outcomes, death many be a censoring event. We will compare the characteristics of the survivors to infants who die using standard descriptive statistics. Rates will be summarized and compared within all infants and within survivors only. Death will be coded as time of censoring in time-to-event outcomes [31].

### Methods in analysis to handle protocol non-adherence and any statistical methods to handle missing data {20c}

A modified “as-randomized” analysis (or “intention-to-treat” analysis) will be used. One pre-specified modification to the analytic set is the exclusion of randomized infants who are not subsequently extubated for new/emergent conditions not known at time of randomization. This number is expected to be minimal and will be closely monitored. All other infants will be included in the analysis under standard ITT assumptions.

### Plans to give access to the full protocol, participant-level data, and statistical code {31c}

We will use CONSORT guidance for reporting results [32]. De-identified participant-level data and code in support of each publication will be made available within 6 months of publication. Limited risk data files will be available at the end of the study under a data use agreement.

## Oversight and monitoring

### Composition of the coordinating center and trial steering committee {5d}

#### The executive committee

The DIVA executive committee consists of the clinical coordinating center (CCC) PIs and DCC PI. This committee assumes ultimate responsibility for the following:

- Ensure study policies and procedures are implemented at study sites
- Determine whether concurrent enrollment in other studies at the sites is acceptable
- Draft protocol amendments when needed
- Liaise at arm’s length with the DSMB and review and act upon DSMB recommendations
- Ensure the rate of enrollment is satisfactory and that this is adhering to the study milestones
- Liaise with the technical committee and steering committee
- Ensure new pertinent literature is disseminated to the steering committee and DSMB
- Approve the final manual of procedures and case report forms (CRFs)
- Monitor trial performance across sites
- Ensure that serious adverse events and adverse events are reported promptly according to IRB and DSMB timelines
- Identify poorly functioning sites and implement remedial procedures and, if needed, remove the site from the trial
- Add new sites if needed to meet enrollment milestones

#### The clinical coordinating center (CCC)

The CCC will direct the clinical aspects of protocol development and study implementation and will be the primary liaison with the site IRBs.

#### The data coordinating center (DCC)

The DCC has statistical operations centered at the UVA and data management operations at the University of Pennsylvania, Philadelphia PA. The DCC will provide statistical collaboration, data management, and information technology support for the development and conduct of the trial. The DCC is responsible for regulatory oversight and coordination of protocol modifications at the participating clinical sites.

#### The technical committee

The DIVA technical committee comprises experts in neonatal respiratory management and, in particular, NAVA. The technical committee will be responsible for developing training materials, training study teams in NIV-NAVA and NS-NIPPV, monitoring study interventions, and providing guidance and troubleshooting questions that arise in ventilator management for all study sites.

#### The steering committee

In addition to the executive and technical committee members, the steering committee will consist of each site PI, two expert advisors with extensive expertise leading neonatal trials of respiratory management, and an NHLBI scientist.

### Composition of the data monitoring committee, its role and reporting structure {21a}

All US sites will use a single IRB (sIRB, University of Pennsylvania), and all reportable events will be submitted to this IRB for initial assessment adhering to the National Institutes of Health (NIH) sIRB policy. International sites will use individual review boards rather than a single IRB. A data safety monitoring board (DSMB) was appointed by the NHLBI (see details below).

The data safety monitoring board (DSMB) is an independent group tasked to provide recommendations to the Office of the Director, NHLBI, including recommendations about starting, continuing, and stopping the study. The DSMB will receive regular reports from the trial on any injuries or adverse events, any developments that jeopardize the continued success of the trial, and data by which to accomplish the evaluation of pre-determined early stopping rules [33].

### Adverse event reporting and harms {22}

Adverse events will be monitored during the study intervention and follow-up phases to ensure timely detection of events that may affect safety or continued participation. Reportable events will be reported to the DCC, who will then ensure all events are reported to the IRB and DSMB.

Pre-specified reportable trial SAEs include:

1. New air leaks (pneumothorax or pulmonary interstitial emphysema)
2. New gastrointestinal perforation or bleeding (theoretical concern from Edi catheter)
3. Death

### Frequency and plans for auditing trial conduct {23}

The executive committee will review sites’ screening logs, protocol violations, and deviations monthly. In the run-in phase, the technical committee will use an assessment tool to review NAVA performance and provide targeted feedback. In addition, the technical committee will audit ventilation management for the first six participants from each site during the randomization phase, regardless of treatment arm. Thereafter, the DCC will assign random technical audits of 10% of infants randomized to both arms throughout the randomization phase.

### Plans for communicating important protocol amendments to relevant parties (e.g., trial participants, ethical committees) {25}

Important protocol modifications (changes to eligibility criteria, outcomes, and analyses) will be promptly communicated to relevant parties. The sponsor and funder have been and will be notified of ethical approval and the clinical register record have been and will be updated.

### Dissemination plans {31a}

At the end of the trial, results will be submitted to the annual Pediatric Academic Societies (PAS) meeting, a high-profile general medical journal, and to ClinicalTrials.gov.

## Discussion

### Synchronizing non-invasive ventilation: testing a novel method

Non-invasive positive pressure support is used to stabilize alveolar recruitment and provide chest wall stability to prevent the need for invasive ventilation and decrease rates of BPD, as demonstrated in observational trials, randomized controlled trials, and meta-analyses [26, 34–39]. Available modes of non-invasive support have included CPAP and NIPPV. CPAP failure is common, and a Cochrane review and meta-analysis have demonstrated that both synchronized and non-synchronized NIPPV are superior to CPAP at preventing extubation failure, but only synchronized NIPPV reduced the incidence of BPD when compared to CPAP [40, 41].

Synchronizing NIPPV in preterm infants is challenging because of the large interface leaks, high respiratory rates, small tidal volumes, and variable breathing patterns inherent to this population. A Graseby pneumatic capsule, which is a small foam-filled disc secured to the abdominal wall to detect tiny pressure changes, has been used but has limitations due to difficulties with capsule positioning and attachment, inaccuracy at fast respiratory rates, motion artifact, and sensitivity-detecting respirations [42–44]. Another option, non-invasive flow triggering, is often inaccurate due to large and fluctuating leaks [45, 46].

NIV-NAVA provides an innovative method to synchronize non-invasive respiratory support with infant respiratory drive. NAVA detects the diaphragm’s electrical activity of the diaphragm (Edi) using a functional naso/orogastric tube with imbedded electrodes and delivers assistance that is synchronized and proportional to the infant’s respiratory drive [47, 48]. Because the neural signal is detected by the Edi catheter in the esophagus, it functions independently of air leaks and motion artifacts, has a rapid response rate, and is therefore synchronous in both invasive and non-invasive ventilation [49].

The neural trigger responds to the infant’s respiratory *drive*, in contrast to flow and pneumatic triggers which respond to the infant’s respiratory *effort*. The neural trigger is synchronous for initiation, size, and termination of the breath but flow and pneumatic triggers are synchronous for breath initiation only. Numerous small studies have demonstrated improved synchrony, lower peak pressures, and improved measures of oxygenation with the use of NAVA in infants [49–65]. However, no adequately powered studies have assessed the impact of NIV-NAVA on clinically relevant outcomes in preterm infants. The DIVA trial will fill this evidence gap.

### Methodological considerations

Three major considerations were encountered in the design of the DIVA trial:

#### Including NAVA-naïve sites

When designing this trial, we considered including clinical sites without NAVA experience. The trial investigators felt it important to investigate whether NAVA is more effective at preventing extubation failure in premature infants across various units. Questions of generalizability would remain if the DIVA trial demonstrated benefit only at centers already expert at using NIV-NAVA. Would the results be generalizable to centers without NAVA experience? The protocol design addresses this by comparing NIV-NAVA to NS-NIPPV for preventing extubation failure and assessing whether NAVA-naïve centers can learn and apply the technology after a relatively brief training period.

#### Ensuring consistent ventilator management

In designing the trial, investigators had to design methods to ensure that all sites were using ventilators in both arms in a consistent fashion. The designers were cognizant of prior struggles in trial conduct, including those encountered during a trial of high-frequency ventilation in a similar population [66]. The following strategies were developed.

1. A study-wide boot camp was conducted at the beginning of the trial for site PIs and study team members.
2. The technical committee provided detailed titration algorithms for both treatment arms.
3. All sites are required to complete the run-in phase, where the technical committee independently assessed technical performance with NIV-NAVA using prespecified and objective criteria.
4. Ongoing audits and assessments of both NAVA and NS-NIPPV management included in the randomization phase to ensure that all trial sites are using the ventilators as intended and consistently.

#### Primary outcome definition

The full rationale for the primary outcome is provided above. The protocol uses a primary outcome of predefined criteria for extubation failure rather than simply reintubation. Extubation failure is a short-term outcome with immediate clinical relevance and is important when considering the role VILI plays in the etiology of BPD. Because the trial is unblinded, there was a concern about performance bias if the outcome of reintubation was used, as there is significant practice variation in decisions to reintubate preterm infants. Therefore, consistent with other high-quality trials in this population, objective clinical physiological criteria are used rather than the practice outcome of reintubation. [26–28, 67]. For these reasons, the composite definition for extubation failure used in this trial overcomes site and provider-level practice variation and reduces the potential for bias.

The duration of time after extubation which constitutes a “successful extubation” is not standardly defined in preterm infants and ranges from 48 h to 7 days [68]. Most reintubations for respiratory indications occur in the first week after extubation, with the highest proportion in the first 5 days [18]. Furthermore, reintubation in the first 48 h is associated with increased odds of BPD, even adjusting for cumulative ventilation days [69]. In the DIVA trial, a 5-day (120-h) window is used to monitor for extubation failure. This was also a pragmatic decision, as the NAVA Edi catheter is designed to be changed after 5 days of use.

## Trial status

This trial is open and enrolling infants using protocol version 1.3, dated January 23, 2003. Recruitment began in August 2022. The approximate date when recruitment will be completed is March 2026.

## Data Availability

The DCC will have access to the final trial dataset. The investigators will comply with all NIH data sharing policies.

## Abbreviations

BPD: Bronchopulmonary dysplasia
CCU: Clinical Research Computing Unit
CPAP: Continuous positive airways pressure
DCC: Data coordinating committee
DMS: Data management system
DSMB: Data safety monitoring board
DIVA: Diaphragmatic Initiated Ventilatory Assist
Edi: Electrical activity of the diaphragm
FDA: Food and Drug Administration
HIPAA: Health insurance portability and accountability act
mITT: Modified intention to treat
NIPPV: Nasal intermittent positive pressure ventilation
NHLBI: National Heart, Lung, and Blood Institute
NIV-NAVA: Non-invasive neurally adjusted ventilatory assist
NIV: Non-invasive ventilation
NS-NIPPV: Non-synchronized nasal intermittent positive pressure ventilation
PPV: Positive pressure ventilation
PMA: Postmenstrual age
PI: Principal investigator
RCT: Randomized controlled trial
UVA: University of Virginia
VILI: Ventilator-induced lung injury

## References

[1] Murray CJL (2013). The state of US health, 1990–2010: burden of diseases, injuries, and risk factors. JAMA.

[2] Patel RM, Kandefer S, Walsh MC, Bell EF, Carlo WA, Laptook AR (2015). Causes and timing of death in extremely premature infants from 2000 through 2011. N Engl J Med.

[3] Doyle LW, Faber B, Callanan C, Freezer N, Ford GW, Davis NM (2006). Bronchopulmonary dysplasia in very low birth weight subjects and lung function in late adolescence. Pediatrics.

[4] Fawke J, Lum S, Kirkby J, Hennessy E, Marlow N, Rowell V (2010). Lung function and respiratory symptoms at 11 years in children born extremely preterm: the EPICure study. Am J Respir Crit Care Med.

[5] Sanchez-Solis M, Garcia-Marcos L, Bosch-Gimenez V, Pérez-Fernandez V, Pastor-Vivero MD, Mondéjar-Lopez P (2012). Lung function among infants born preterm, with or without bronchopulmonary dysplasia. Pediatr Pulmonol.

[6] Vollsæter M, Røksund OD, Eide GE, Markestad T, Halvorsen T (2013). Lung function after preterm birth: development from mid-childhood to adulthood. Thorax.

[7] Wang L-YY, Luo H-JJ, Hsieh W-SS, Hsu C-HH, Hsu H-CC, Chen P-SS (2010). Severity of bronchopulmonary dysplasia and increased risk of feeding desaturation and growth delay in very low birth weight preterm infants. Pediatr Pulmonol..

[8] Schmidt B, Asztalos EV, Roberts RS, Robertson CMT, Sauve RS, Whitfield MF (2003). Impact of bronchopulmonary dysplasia, brain injury, and severe retinopathy on the outcome of extremely low-birth-weight infants at 18 months: results from the trial of indomethacin prophylaxis in preterms. JAMA.

[9] Ehrenkranz RA, Walsh MC, Vohr BR, Jobe AH, Wright LL, Fanaroff AA (2005). Validation of the National Institutes of Health consensus definition of bronchopulmonary dysplasia. Pediatr.

[10] Short EJJ, Klein NKK, Lewis BAA, Fulton S, Eisengart S, Kercsmar C (2003). Cognitive and academic consequences of bronchopulmonary dysplasia and very low birth weight: 8-year-old outcomes. Pediatr.

[11] Natarajan G, Pappas A, Shankaran S, Kendrick DE, Das A, Higgins RD (2012). Outcomes of extremely low birth weight infants with bronchopulmonary dysplasia: impact of the physiologic definition. Early Hum Dev.

[12] Johnson TJ, Patel AL, Jegier BJ, Engstrom JL, Meier PP (2013). Cost of morbidities in very low birth weight infants. J Pediatr..

[13] Bhandari A, Carroll C, Bhandari V. BPD following preterm birth: a model for chronic lung disease and a substrate for ARDS in childhood. Front Pediatr Internet. 2016 cited 2019;4. Available from: https://www.frontiersin.org/articles/10.3389/fped.2016.00060/full. 10.3389/fped.2016.00060 PMC4908128 27379219

[14] Jobe AH (2011). The new bronchopulmonary dysplasia. Curr Opin Pediatr.

[15] Walsh MC, Morris BH, Wrage LA, Vohr BR, Poole WK, Tyson JE (2005). Extremely low birthweight neonates with protracted ventilation: mortality and 18-month neurodevelopmental outcomes. J Pediatr.

[16] Laughon MM, Langer JC, Bose CL, Smith PB, Ambalavanan N, Kennedy KA (2011). Prediction of bronchopulmonary dysplasia by postnatal age in extremely premature infants. Am J Respir Crit Care Med.

[17] Chawla S, Natarajan G, Shankaran S, Carper B, Brion LP, Keszler M (2017). Markers of successful extubation in extremely preterm infants, and morbidity after failed extubation. J Pediatr.

[18] Shalish W, Kanbar L, Keszler M, Chawla S, Kovacs L, Rao S (2018). Patterns of reintubation in extremely preterm infants: a longitudinal cohort study. Pediatr Res.

[19] Kirpalani H, Millar D, Lemyre B, Yoder BA, Chiu A, Roberts RS (2013). A trial comparing non-invasive ventilation strategies in preterm infants. N Engl J Med.

[20] Jensen EA, DeMauro SB, Kornhauser M, Aghai ZH, Greenspan JS, Dysart KC (2015). Effects of multiple ventilation courses and duration of mechanical ventilation on respiratory outcomes in extremely low-birth-weight infants. JAMA Pediatr.

[21] Foglia EE, Ades A, Sawyer T, Glass KM, Singh N, Jung P (2019). Neonatal intubation practice and outcomes: an international registry study. Pediatrics.

[22] Thomas RE, Rao SC, Minutillo C, Vijayasekaran S, Nathan EA (2018). Severe acquired subglottic stenosis in neonatal intensive care graduates: a case–control study. Archives of Disease in Childhood - Fetal and Neonatal Edition.

[23] Ferguson KN, Roberts CT, Manley BJ, Davis PG (2017). Interventions to improve rates of successful extubation in preterm infants: a systematic review and meta-analysis. JAMA Pediatr.

[24] Committee on Fetus and Newborn (2014). Respiratory support in preterm infants at birth. Pediatr.

[25] Cummings JJ, Polin RA, the COMMITTEE ON FETUS AND NEWBORN, Watterberg KL, Poindexter B, Cummings JJ, Benitz WE, Eichenwald EC, Poindexter BB, Stewart DL, Aucott SW, Goldsmith JP, Puopolo KM, Wang KS. Noninvasive Respiratory Support. Pediatrics. 2016;137(1):e20153758. 10.1542/peds.2015-3758. 10.1542/peds.2015-3758 26715607

[26] Finer NN, Carlo WA, Walsh MC, Rich W, Gantz MG, Laptook AR (2010). Early CPAP versus surfactant in extremely preterm infants. N Engl J Med.

[27] Roberts CT, Owen LS, Manley BJ, Frøisland DH, Donath SM, Dalziel KM (2016). Nasal high-flow therapy for primary respiratory support in preterm infants. N Engl J Med.

[28] Manley BJ, Arnolda GRB, Wright IMR, Owen LS, Foster JP, Huang L (2019). Nasal high-flow therapy for newborn infants in special care nurseries. N Engl J Med..

[29] Jensen EA, Dysart K, Gantz MG, McDonald S, Bamat NA, Keszler M, Kirpalani H, Laughon MM, Poindexter BB, Duncan AF, Yoder BA, Eichenwald EC, DeMauro SB. The Diagnosis of Bronchopulmonary Dysplasia in Very Preterm Infants. An Evidence-based Approach. Am J Respir Crit Care Med. 2019;200(6):751–9. 10.1164/rccm.201812-2348OC. 10.1164/rccm.201812-2348OC PMC6775872 30995069

[30] Makker K, Cortez J, Jha K, Shah S, Nandula P, Lowrie D (2020). Comparison of extubation success using non-invasive positive pressure ventilation (NIPPV) versus non-invasive neurally adjusted ventilatory assist (NI-NAVA). J Perinatol.

[31] Harhay MO, Ratcliffe SJ, Small DS, Suttner LH, Crowther MJ, Halpern SD (2019). Measuring and analyzing length of stay in critical care trials. Med Care.

[32] Schulz KF, Altman DG, Moher D, CONSORT Group. CONSORT (2010). statement: updated guidelines for reporting parallel group randomised trials. BMJ.

[33] Lan KKG, DeMets DL (1983). Discrete sequential boundaries for clinical trials. Biometrika.

[34] Avery ME, Tooley WH, Keller JB, Hurd SS, Bryan MH, Cotton RB (1987). Is chronic lung disease in low birth weight infants preventable?. A survey of eight centers Pediatr.

[35] Morley CJ, Davis PG, Doyle LW, Brion LP, Hascoet J-MM, Carlin JB (2008). Nasal CPAP or intubation at birth for very preterm infants. N Engl J Med..

[36] Dunn MS, Kaempf J, de Klerk A, de Klerk R, Reilly M, Howard D (2011). Randomized trial comparing 3 approaches to the initial respiratory management of preterm neonates. Pediatr.

[37] Schmölzer GM, Kumar M, Pichler G, Aziz K, O’Reilly M, Cheung PY (2013). Non-invasive versus invasive respiratory support in preterm infants at birth: systematic review and meta-analysis. BMJ.

[38] Fischer HS, Bührer C (2013). Avoiding endotracheal ventilation to prevent bronchopulmonary dysplasia: a meta-analysis. Pediatr.

[39] Subramaniam P, Ho JJ, Davis PG (2016). Prophylactic nasal continuous positive airway pressure for preventing morbidity and mortality in very preterm infants. Cochrane Database Syst Rev..

[40] Dargaville PA, Gerber A, Johansson S, De Paoli AG, Kamlin COF, Orsini F (2016). Incidence and outcome of CPAP failure in preterm infants. Pediatr..

[41] Lemyre B, Davis PG, De Paoli AG, Kirpalani H (2017). Nasal intermittent positive pressure ventilation (NIPPV) versus nasal continuous positive airway pressure (NCPAP) for preterm neonates after extubation. Cochrane Database Syst Rev..

[42] Stern DJ, Weisner MD, Courtney SE (2014). Synchronized neonatal non-invasive ventilation-a pilot study: the Graseby capsule with bi-level NCPAP. Pediatr Pulmonol.

[43] Chang H-Y, Claure N, D’ugard C, Torres J, Nwajei P, Bancalari E (2011). Effects of synchronization during nasal ventilation in clinically stable preterm infants. Pediatr Res..

[44] Ramos-Navarro C, Sanchez-Luna M, Sanz-López E, Maderuelo-Rodriguez E, Zamora-Flores E (2016). Effectiveness of synchronized noninvasive ventilation to prevent intubation in preterm infants. AJP Rep.

[45] Gizzi C, Montecchia F, Panetta V, Castellano C, Mariani C, Campelli M (2015). Is synchronised NIPPV more effective than NIPPV and NCPAP in treating apnoea of prematurity (AOP)? A randomised cross-over trial. Arch Dis Child Fetal Neonatal Ed.

[46] Courtney SE, Barrington KJ (2007). Continuous positive airway pressure and non-invasive ventilation. Clin Perinatol..

[47] Eichenwald EC, Howell RG, Kosch PC, Ungarelli RA, Lindsey J, Stark R (1992). Developmental changes in sequential activation of laryngeal abductor muscle and diaphragm in infants. J Appl Physiol.

[48] Sinderby C, Navalesi P, Beck J, Skrobik Y, Comtois N, Friberg S (1999). Neural control of mechanical ventilation in respiratory failure. Nat Med.

[49] Beck J, Reilly M, Grasselli G, Mirabella L, Slutsky AS, Dunn MS (2009). Patient-ventilator interaction during neurally adjusted ventilatory assist in low birth weight infants. Pediatr Res.

[50] Stein H, Howard D. Neurally adjusted ventilatory assist in neonates weighing <1500 grams: a retrospective analysis. J Pediatr. 2012;160(5):786–9.e1. 10.1016/j.jpeds.2011.10.014. 10.1016/j.jpeds.2011.10.014 22137670

[51] Stein H, Alosh H, Ethington P, White DB (2013). Prospective cross-over comparison between NAVA and pressure control ventilation in premature neonates less than 1500 grams. J Perinatol.

[52] Lee J, Kim H-S, Sohn JA, Lee JA, Choi CW, Kim E-K (2012). Randomized cross-over study of neurally adjusted ventilatory assist in preterm infants. J Pediatr.

[53] Chen Z, Luo F, Ma XL, Lin HJ, Shi LP, Du LZ (2013). Application of neurally adjusted ventilatory assist in preterm infants with respiratory distress syndrome. Zhongguo Dang Dai Er Ke Za Zhi.

[54] Longhini F, Ferrero F, Luca DD, Cosi G, Alemani M, Colombo D (2015). Neurally adjusted ventilatory assist in preterm neonates with acute respiratory failure. NEO.

[55] Lee J, Kim H-S, Jung YH, Shin SH, Choi CW, Kim E-K (2015). Non-invasive neurally adjusted ventilatory assist in preterm infants: a randomised phase II cross-over trial. Arch Dis Child Fetal Neonatal Ed.

[56] Firestone KS, Fisher S, Reddy S, White DB, Stein HM (2015). Effect of changing NAVA levels on peak inspiratory pressures and electrical activity of the diaphragm in premature neonates. J Perinatol.

[57] LoVerde B, Firestone KS, Stein HM (2016). Comparing changing neurally adjusted ventilatory assist (NAVA) levels in intubated and recently extubated neonates. J Perinatol.

[58] Kallio M, Koskela U, Peltoniemi O, Kontiokari T, Pokka T, Suo-Palosaari M (2016). Neurally adjusted ventilatory assist (NAVA) in preterm newborn infants with respiratory distress syndrome—a randomized controlled trial. Eur J Pediatr.

[59] Shetty S, Hunt K, Peacock J, Ali K, Greenough A (2017). Cross-over study of assist control ventilation and neurally adjusted ventilatory assist. Eur J Pediatr.

[60] Colaizy TT, Kummet GJ, Kummet CM, Klein JM (2017). Non-invasive neurally adjusted ventilatory assist in premature infants postextubation. Amer J Perinatol.

[61] Yonehara K, Ogawa R, Kamei Y, Oda A, Kokubo M, Hiroma T (2018). Non-invasive neurally adjusted ventilatory assist versus nasal intermittent positive-pressure ventilation in preterm infants born before 30 weeks’ gestation. Pediatr Int.

[62] Lee BK, Shin SH, Jung YH, Kim EK, Kim HS (2019). Comparison of NIV-NAVA and NCPAP in facilitating extubation for very preterm infants. BMC Pediatr.

[63] Kallio M, Mahlman M, Koskela U, Aikio O, Suo-Palosaari M, Pokka T (2019). NIV NAVA versus nasal CPAP in premature infants: a randomized clinical trial. Neonatology.

[64] Yagui AC, Gonçalves PA, Murakami SH, Santos AZ, Zacharias RSB, Rebello CM (2021). Is noninvasive neurally adjusted ventilatory assistance (NIV-NAVA) an alternative to NCPAP in preventing extubation failure in preterm infants?. J Matern Fetal Neonatal Med.

[65] Firestone K, Horany BA, de Leon-Belden L, Stein H (2020). Nasal continuous positive airway pressure versus noninvasive NAVA in preterm neonates with apnea of prematurity: a pilot study with a novel approach. J Perinatol.

[66] Courtney SE, Durand DJ, Asselin JM, Hudak ML, Aschner JL (2002). Shoemaker CT; Neonatal Ventilation Study Group. High-frequency oscillatory ventilation versus conventional mechanical ventilation for very-low-birth-weight infants. N Engl J Med.

[67] Kirpalani H, Ratcliffe SJ, Keszler M, Davis PG, Foglia EE, te Pas A (2019). Effect of sustained inflations vs intermittent positive pressure ventilation on bronchopulmonary dysplasia or death among extremely preterm infants: the SAIL randomized clinical trial. JAMA.

[68] Giaccone A, Jensen E, Davis P, Schmidt B (2014). Definitions of extubation success in very premature infants: a systematic review. Arch Dis Child Fetal Neonatal Ed.

[69] Shalish W, Kanbar L, Kovacs L, Chawla S, Keszler M, Rao S (2019). The impact of time interval between extubation and reintubation on death or bronchopulmonary dysplasia in extremely preterm infants. J Pediatr.

